# Vinflunine in routine clinical practice for the treatment of advanced or metastatic urothelial cell carcinoma - data from a prospective, multicenter experience

**DOI:** 10.1186/s12885-015-1434-3

**Published:** 2015-06-04

**Authors:** Margitta Retz, Patrick de Geeter, Peter J. Goebell, Ullrich Matz, Wito de Schultz, Axel Hegele

**Affiliations:** 1 Urologische Klinik und Poliklinik, Technische Universität München, Ismaninger Str. 22, 81675 Munich, Germany; 2 Department of Urology, Kassel Hospital, Kassel, Germany; 3 Department of Urology, Friedrich-Alexander University, Erlangen, Germany; 4 Urologic practice, Doebeln, Germany; 5 Medical Urology practice, Leipzig, Germany; 6 Department of Urology / Pediatric Urology, Philipps University, Marburg, Germany

**Keywords:** Advanced or metastatic urothelial cell carcinoma, Routine clinical practice, Vinflunine

## Abstract

**Background:**

Vinflunine is recommended in the European guideline for the treatment of advanced or metastatic urothelial cell carcinoma (UCC) after failure of platinum-based therapy.

**Methods:**

This prospective, non-interventional study investigated the safety and efficacy of vinflunine in platinum-pretreated UCC patients in routine clinical practice. Data were prospectively collected on patients with advanced or metastatic UCC undergoing vinflunine treatment in 39 German hospitals and medical practices. Dosing of vinflunine, tumor assessments and concomitant medications followed physician’s routine clinical practice. Primary endpoints were toxicity and assessment of vinflunine treatment modalities. Secondary aims included overall response rate (ORR), overall survival (OS) time and a prognostic risk-model.

**Results:**

Seventy-seven platinum-pretreated patients were recruited. Vinflunine was predominantly administered as second-line (66 %) therapy or in subsequent treatment lines (21 %). One third of the patients received at least six cycles of vinflunine and the average number was 4.7 cycles. A vinflunine starting dose of 320 mg/m^2^ was chosen in 48 % of patients and 280 mg/m^2^ in 39 %. Grade 3/4 toxicities were leucopenia 16.9 %, anemia 6.5 %, elevated liver enzymes 6.5 % and constipation 5.2 %. ORR was 23.4 % and OS was 7.7 (CI 4.1 to 10.4) months. Patients with zero, one, two or ≥ three risk factors displayed a median OS of 18.2, 9.5, 4.1 and 2.8 months, respectively (*p* = 0.0005; HR = 1.82).

**Conclusion:**

Vinflunine delivers a meaningful benefit to an unselected population of advanced platinum-pretreated UCC patients managed in routine clinical practice.

## Background

Metastatic urothelial cell carcinoma (UCC) responds well to chemotherapy. With cisplatin-based regimens a median overall survival (OS) of 12.5–15.5 months can be reached in the first-line setting [1, 2]. However, there were limited therapeutic options for patients who subsequently failed cisplatin-based therapy.

When vinflunine was approved by the European Medicines Agency (EMA) in 2009, it was the first chemotherapy to be registered for use following failure of platinum-based treatment. Vinflunine is a novel microtubule inhibitor and has shown improved patient outcomes in a multicenter, placebo-controlled phase III trial [3]. In this study, 370 patients with metastatic UCC who failed first-line platinum-based chemotherapy were randomized 2:1 to receive either vinflunine plus best supportive care (BSC) or BSC alone. In the intent-to-treat (ITT) population median OS was 6.9 months and 4.6 months in the vinflunine plus BSC and BSC arm, respectively. Though this difference did not reach statistical significance vinflunine treatment correlated with increased survival [4]. The final analysis on the eligible population demonstrated a median OS of 6.9 months for the vinflunine group versus 4.3 months for the BSC arm, showing an estimated 22 % reduction in the risk of death (*p* = 0.0227). Overall response rate (ORR), disease control rate (DCR), and progression free survival (PFS) were all statistically significant in favor of vinflunine [3]. Subsequently vinflunine has been recommended as the standard of care in these patients in the EAU guidelines [5].

Although vinflunine was efficacious in the randomized phase III trial, patients under study conditions may not fully reflect actual clinical practice as they are selected and their therapy management is strictly protocol-driven. Consequently, the use of vinflunine in routine clinical practice might not fully translate into similar outcomes to the registration study data.

The objective of this observational study was to examine the efficacy and toxicity of vinflunine as well as the adverse events (AE) management in routine clinical practice, where unselected patients were treated following the registered marketing authorization across a prospective, multicenter and non-interventional study (NIS). Furthermore, a prognostic risk-model was evaluated for the NIS population.

## Methods

In compliance with the German Drug Law (AMG) the non-interventional study was reported to the competent authority and approved by the ethics committee of the scientific leader (ethics committee of the Technische Universitaet München, Germany). The prospective NIS included patients with histologically confirmed locally advanced or metastatic UCC who experienced failure of a prior platinum-based chemotherapy. Patients had to have an Eastern Cooperative Oncology Group (ECOG) performance status (PS) of 0 or 1, as well as an adequate hematologic, hepatic and renal (calculated clearance of creatinine > 20 ml/min) function. Main exclusion criteria were brain metastases and a life expectancy < 2 months. A total of 77 patients were enrolled from 15 urological and 3 oncological hospitals (42 and 6 patients, respectively) as well as from 14 urological and 7 oncological practices (20 and 9 patients, respectively) throughout Germany. Initially it was planned to recruit 200 patients but the study was discontinued before reaching the planned sample size due to slow recruitment. The NIS was conducted according to the provisions as laid down in the Declaration of Helsinki and registered on clinicaltrials.gov (NCT01103544). All patients were required to sign written informed consent before any documentation of patient data could take place and the decision to treat with vinflunine had to be drawn independently from the study (i.e. before the decision to participate in this NIS).

Owing to the non-interventional design of this trial, physicians were not instructed on any treatment decisions including dosages of vinflunine, tumor assessments, AE-management and concomitant medications. An antiemetic- and laxative prophylaxis was recommended in accordance to the routine clinical practice. The planned observation period was limited to a maximum of six cycles of vinflunine. The patient's final documentation took place 30 days after the last administration of vinflunine or after the sixth administration. Data were prospectively collected in standardized electronic case report forms on patient characteristics (performance status [6]), AE according to the National Cancer Institute Common Terminology Criteria for Adverse Events (NCI-CTCAE) v3 criteria, vinflunine dosages and number of administered cycles. Furthermore, tumor assessments and co-medication or dietary measures to prevent constipation and nausea were documented. Patients were additionally followed for survival information. Missing or inconsistent data were identified by central data review and clarified by corresponding data queries.

Primary endpoints of this study were the frequency of AE according to the NCI-CTCAE v3 criteria as well as the assessment of vinflunine treatment modalities (e.g. dosage, duration, concomitant medication) with the aim of a descriptive analysis. Secondary aims included the ORR and the median OS. The protocol suggested that the tumor response is assessed at least once during treatment (i.e. between the first chemotherapy visit and the final visit) by imaging and evaluated according to the RECIST 1.1 criteria. Additionally, prognostic factors for advanced UCC receiving second-line systemic therapy were evaluated based on the prognostic stratification model by Sonpavde et al. [7] The model defines four risk factors: liver metastases, ECOG PS, hemoglobin (Hb) value and time from prior chemotherapy (TFPC).

Explorative methods were used for the analysis of the collected data. All collected parameters were analyzed descriptively. Arithmetic means, standard deviations and 95 % confidence intervals (CI) were calculated for continuous characteristics. Differences in baseline parameters were tested by one-way ANOVA (analysis of variance). Time-based efficacy parameters were presented as Kaplan-Meier curves and subject to the log-rank test for significance. For the risk factor model analysis, patients were assigned to the lower risk group if values were missing to perform the analysis on the full patient population (statistical significance was confirmed by leaving out patients with missing values). Statistical analysis was performed on the basis of SAS version 9.2.

## Results

From 08/2010 to 09/2011, 77 patients were evaluable on an intent-to-treat (ITT) basis from 39 German centers. The median age of the ITT group was 67 (range 39–80) years (Table 1). An ECOG PS0 and PS1 was present in 45.5 % and 54.5 % of all patients, respectively. Visceral involvement was found in 59.7 % of patients.

**Table 1:** Patient demographics and clinical characteristics (ITT-population: *n* = 77)

	Overall	Starting dose	Starting dose
		320 mg/m^2^	≤280 mg/m^2^
	n = 77	n = 37	n = 40 *
Female	14 (18.2 %)	6 (16.2 %)	8 (20.0 %)
Male	63 (81.1 %)	31 (83.3 %)	32 (80.0 %)
Median age in years (range)	67 (39–80)	66 (39–77)	70 (51–80)
≥70 y	32 (41.6 %)	12 (32.4 %)	20 (50.0 %)
ECOG PS			
0	35 (45.5 %)	18 (48.6 %)	17 (42.5 %)
1	42 (54.5 %)	19 (51.4 %)	23 (57.5 %)
Visceral involvement	46 (59.7 %)	23 (62.2 %)	23 (57.5 %)
Liver metastases^†^	17 (22.1 %)	6 (16.2 %)	11 (27.5 %)
TFPC < 6 months^‡^	45 (58.4 %)	19 (51.4 %)	26 (65.0 %)
Mean Hb in g/dL^§^	11.2	11.6	10.9
Hb < 10 g/dL^§^	13 (16.9 %)	5 (13.5 %)	8 (20.0 %)

ECOG: Eastern Cooperative Oncology Group; PS: performance status; TFPC: time from prior chemotherapy; Hb: hemoglobin

*n(280 mg/m^2^) = 30; n(<280 mg/m^2^) = 10

^†^information missing for 4 patients in the 320 mg/m^2^ group and 8 patients in the ≤ 280 mg/m^2^ group

^‡^information missing for 6 patients in the 320 mg/m^2^ group and 2 patients in the ≤ 280 mg/m^2^ group

^§^information missing for 6 patients in the 320 mg/m^2^ group and 2 patients in the ≤ 280 mg/m^2^ group; ITT: intent-to-treat

**Fig. 1:**
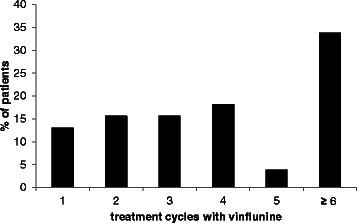
Number of vinflunine treatment cycles (ITT- population: *n* = 77); documentation was limited to a maximum of six vinflunine treatment cycles

Previous platinum-based chemotherapy was administered in (neo)-adjuvant or palliative intentions. Accordingly, vinflunine was scheduled as first line treatment for 12 % of the patients following (neo)-adjuvant platinum-based regimens. Notably, vinflunine was predominantly administered as second-line palliative therapy in 66 % of all patients, as third-line in 18 % and as further treatment line in 3 %. Focusing on different pre-treatment schedules, 82 % of all patients were pretreated with the combination of gemcitabine and cisplatin, 12 % with gemcitabine and carboplatin and 12 % received gemcitabine and paclitaxel (Table 2). Monotherapy with different antineoplastic agents such as gemcitabine, cisplatin or paclitaxel was performed in 17 % of the study group. About half of the patients were treated with a starting dose of 320 mg/m^2^ and the other half with 280 mg/m^2^ or less. Patients with the higher starting dose were younger (*p* = 0.03) (Table 1). The median number of vinflunine cycles was four (mean 4.7) cycles and one-third of the patients received at least six cycles (Fig. 1). A concomitant antiemetic therapy was given in 65 patients (84.4 %). The most commonly used agents were dexamethasone (46.2 %), granisetron (44.6 %) and/or metoclopramide (23.1 %); these were predominantly given in a prophylactic intent (95.4 %). Co-medication to prevent constipation was administered in 55 patients (71.4 %). Commonly used laxative agents were macrogol (65.5 %) and/or lactulose (14.5 %), usually given prophylactically (87.3 %).

**Table 2:** Treatment regimens prior to vinflunine (ITT-population: *n* = 77)

Previous treatment	% patients
Gemcitabine + cisplatin	82
Gemcitabine + carboplatin	12
Gemcitabine + paclitaxel	12
Gemcitabine	9
Cisplatin	4
Paclitaxel	4
Other regimens	14

Patients might have had more than one previous therapy. Therapies were listed if > 3 % of the patients were treated with the regimen. Other regimens include (with decreasing frequency): methotrexate/vinblastine/doxorubicin/cisplatin (conventional and dose dense), docetaxel, cisplatin/paclitaxel, carboplatin/paclitaxel

A total of 272 AEs were documented in 55 (71.4 %) patients, of which 177 AEs were assessed as potentially related to vinflunine. About half of the patients (49.4 %) experienced at least one AE considered to be related to vinflunine. The most frequent treatment-related AEs considering all grades of intensity were hematological toxicities (Table 3): leucopenia (22.1 %), anemia (16.9 %) and thrombocytopenia (7.8 %). Hematological adverse events of grade ≥ 3 mainly included leucopenia (16.9 %) and anemia (6.5 %). Only one patient (1.3 %) experienced neutropenic infection. The most commonly reported non-hematological toxicities grade ≥ 3 were elevated liver enzymes (6.5 %) and constipation (5.2 %).

**Table 3:** Most common treatment-related adverse events (ITT-population: *n* = 77)

	All grades		Grades ≥ 3^*^	
	N	%	N	%
Any event	38	49.4	23	29,9
Leukopenia	17	22.1	13	16.9
Anemia	13	16.9	5	6.5
Thrombocytopenia	6	7.8	1	1.3
Neutropenia	2	2.6	1	1.3
Elevated liver enzymes^‡^	16	20.8	5	6.5
Fatigue	12	15.6	1	1.3
Pain^§^	10	13.0	3	3.9
Constipation	9	11.7	4	5.2
Nausea	9	11.7	2	2.6
Constitutional symptoms	4	5.2	3	3.9
Infection^†^	3	3.9	3	3.9
Mucositis	3	3.9	0	0.0
Vomiting	2	2.6	2	2.6
Neutropenic infection	1	1.3	1	1.3

Adverse events occurring in at least 3 patients are listed. For completeness neutropenia and vomiting have been added

^*^3 potentially treatment-related deaths were reported: infection (relation possible), infection (relation likely), hypoxia (relation possible)

^‡^Summarizing elevated alanine-aminotransferase (ALT), aspartate-aminotransferase (AST), alkaline phosphatase and gamma-glutamintranferase (GGT)

^§^Pain in abdomen, extremity, head, joint or stomach

^†^Infections with normal absolute neutrophil count (ANC)

**Fig. 2:**
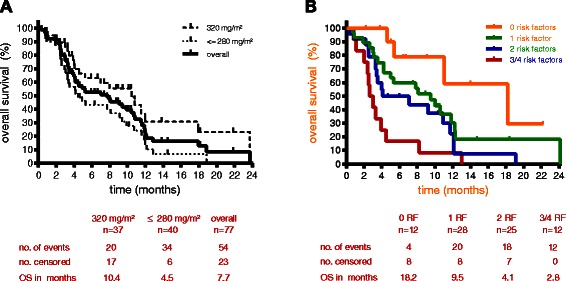
Overall survival (ITT: intent-to-treat OS: overall survival-population: *n* = 77); (**a**) of the overall population (solid line), patients with a starting dose of 320 mg/m^2^ (broken line) and ≤ 280 mg/m^2^ (dotted line); (**b**) according to prognostic factors defined by Sonpavde et al.; overall survival is displayed according to the number of risk factors: 0 (orange), 1 (green), 2 (blue), 3/4 (red) risk factors; risk factors were: presence of liver metastasis, ECOG = 1, Hb value < 10 g/dL, time from prior chemotherapy < 6 months; RF: risk factor; ITT: intent-to-treat; OS: overall survival

To evaluate the ORR, 72 patients (93.5 %) underwent at least one tumor assessment (median 2, range 1–7) by imaging methods. The ORR was 23.4 % (95 % CI, 14.5 % to 34.4 %). In total, four of 77 patients (5.2 %) achieved complete response and 14 (18.2 %) experienced partial response. More than half of the patients (53.2 %) reached disease control (DC) during treatment with vinflunine (Table 4). Investigating survival data, 54 of 77 patients (70.1 %) died within the study period. Median observation time was 4.6 (0.4–23.8) months. The median OS time was 7.7 months (95 % CI, 4.1 months to 10.4 months) (Fig. 2A). Stratifying OS by vinflunine starting dose, patients receiving the recommended vinflunine dose of 320 mg/m^2^ achieved significantly longer median OS with 10.4 months compared to patients treated with starting doses ≤ 280 mg/m^2^ with a median OS of 4.5 months (p = 0.016).

**Table 4:** Efficacy results (ITT: intent-to-treat-population: *n* = 77)

	No. of patients	%	95 % CI, %
Complete response	4	5.2	
Partial response	14	18.2	
Stable disease	23	29.9	
Objective response rate	18	23.4	14.5-34.4
Disease control rate	41	53.2	41.5-64.7

Data from the NIS were analyzed using the risk factor model established by Sonpavde and coworkers [7]. This model defines four risk factors; presence of liver metastases, ECOG PS = 1, mean Hb value < 10 g/dL and TFPC < 6 months. Median OS inversely correlated with the number of risk factors: patients with zero, one, two or ≥ three risk factors displayed a median OS time of 18.2 months, 9.5 months, 4.1 months and 2.8 months, respectively (*p* = 0.0005; HR = 1.82) (Fig. 2B). Both presence of liver metastases and ECOG PS 1 were independent prognostic factors for shorter survival in a univariable analysis (HR =4.330, *p* < 0.0001 and HR = 1.820, *p* = 0.0310, respectively). In a multivariate analysis, the presence of liver metastases was the strongest risk factor influencing OS (*p* < 0.0001).

## Discussion

Non-interventional studies (NIS) are highly valuable for guiding clinical practice. On the one hand they may more accurately reflect the efficacy and safety of treatment strategies under “real world” conditions; on the other hand NIS may give insights into how therapies might be applied more effectively. Although NIS provide useful supplementary information, they cannot substitute for the data from randomized controlled trials. The lack of control over patient management, the specification of interventions as well as timing and type of tumor assessments results in the facts that NIS cannot deliver precise progression-free-survival data, efficacy data must be interpreted with caution and, furthermore, adverse effects might be underestimated due to less frequent visit procedures; nevertheless severe AEs are usually reported.

Despite the limitations inherent in NIS, this trial clearly demonstrates that the efficacy and safety of vinflunine therapy - as observed in controlled, randomized studies - is transferable in the daily routine of clinical practice [3, 8, 9]. Particularly the feasibility of vinflunine treatment is not limited to hospitals since more than one third of the NIS patients were treated in medical practices. The high numbers and the diversity of recruiting centers indicate that vinflunine treatment is manageable in various and different settings.

Although vinflunine is approved by the EMA as chemotherapy after failure of platinum-based treatment, 20 % of the patients were scheduled for third- and fourth-line therapy. Nevertheless, the majority of the patients (66 %) received vinflunine as second-line palliative treatment, which is comparable to the French observational study by Medioni and coworkers [10]^.^

Considering that a maximum of six cycles was protocolled in this study, the average cycle number of 4.7 cycles and the fact that one-third of the patients received at least six cycles suggests a good tolerability of vinflunine. Furthermore, 48.1 % and 39.0 % of the NIS patients received a vinflunine starting dose of 320 mg/m^2^ or 280 mg/m^2^, respectively. These data confirm an equivalent study drug exposure as found in the phase-III trial by Bellmunt and coworkers [3].

One primary endpoint of this study was the frequency of adverse events according to the NCI-CTCAE v3. Hematological toxicity was low in the NIS population with grade 3/4 neutropenia of 1.3 % and leucopenia 16.9 % in contrast to 50 % neutropenia in the randomized controlled phase III study [3]. Retrospective multicenter studies performed in Spain and France displayed incidences of 12.8 % and 17.2 % [11, 10], respectively. Notably, neutropenic infections were reported in 1.3 % of the NIS patients compared to 6.0 % in the trial from Bellmunt and coworkers [3]. The incidence of gastrointestinal toxicities was low with grade 3/4 constipation in 5.2 % and nausea and vomiting each in 2.6 % of the patients. These low gastrointestinal toxicities were possibly achieved because more than 84 % and 71 % of the NIS patients received the recommended antiemetic and laxative prophylaxis, respectively. The gastrointestinal tolerability was slightly better compared to the results of the prospective phase-III trial. The incidence of constipation of 11.7 % was in line with the data of the Spanish and French retrospective studies [10, 11]. These observations across three countries could suggest that an increased experience in the drug use could play a role in the safety findings with physicians learning how to optimize side effect prophylaxis in routine practice.

The efficacy results in the NIS trial are consistent with the previous data of the prospective phase III study [3]. About one-quarter of the patients (23.4 %) responded to vinflunine treatment. More than half of the patients (53.2 %) reached at least DC, which seems to be a representative result compared to other vinflunine trials with a DC rate of 41.1 % in the randomized phase III trial as well as 51 % and 65.7 % in both retrospective studies [3, 10, 11]. The median OS time was 7.7 months for the whole NIS group, which is slightly higher compared to the survival results from the phase III trial with 6.9 months [3, 4]. Similar results have been found in retrospective studies with a median OS time between 8.1 months and 10.0 months [10–12]. Of note, a vinflunine starting dose of 320 mg/m^2^ resulted in a better outcome regarding median OS time (10.4 months) compared to starting doses of ≤ 280 mg/m^2^ (4.5 months). The population was younger in the 320 mg/m^2^ starting dose group compared to the group with lower initial vinflunine doses (median age 66 vs. 70 years, respectively). Patients with lower vinflunine starting dose tended to have more risk factors at baseline (liver metastases, TPFC, Hb < 10 g/dl) though the difference was not significant. In summary, the NIS analysis strongly recommends an optimal vinflunine starting dose of 320 mg/m^2^ as long as there are no medical restrictions.

Data from this NIS were analyzed according to the prognostic risk factor model from Sonpavde and coworkers [7]. On the basis of pooled data from seven prospective phase II trials of patients with advanced platinum-pretreated UCC, the risk factor model stratified four prognostic factors including Hb level, performance score (ECOG-PS), presence of liver metastases and TFPC. The analysis from Sonpavde et al. [7] reported a median OS of 12.2 months, 6.7 months, 5.1 months and 3.0 months for patients with zero, one, two and three to four risk factors, respectively. Our non-interventional study resulted in similar differences in median OS times with 18.2 months, 9.5 months, 4.1 months and 2.8 months for the respective risk factor group, confirming the validity of the data collected under routine clinical conditions. The risk factor model could help to define the individual’s prognosis more accurately. Nevertheless, individual patients with multiple risk factors could still benefit from vinflunine treatment. Furthermore, the model may improve the interpretation and setup of clinical trials.

## Conclusion

This prospective non-interventional study confirmed that vinflunine delivers a meaningful benefit to an unselected population of advanced platinum-pretreated UCC patients managed in routine clinical practice. A systematic gastrointestinal prophylaxis is strongly recommended to achieve a good safety profile. The vinflunine starting dose of 320 mg/m^2^ was most efficacious with a median OS of 10.4 months and should therefore be considered in all eligible patients. This study adds further support to the EAU recommendation for the use of vinflunine as second-line therapy in advanced UCC after failure of platinum-based treatment.
